# Hospitalization costs among immobile patients with hemorrhagic or ischemic stroke in China: a multicenter cross-sectional study

**DOI:** 10.1186/s12913-020-05758-6

**Published:** 2020-09-29

**Authors:** Hongpeng Liu, Chen Zhu, Jing Cao, Jing Jiao, Baoyun Song, Jingfen Jin, Yilan Liu, Xianxiu Wen, Shouzhen Cheng, Xinjuan Wu

**Affiliations:** 1 Department of Nursing, Chinese Academy of Medical Sciences - Peking Union Medical College, Peking Union Medical College Hospital, 1 Shuaifuyuan, Dongcheng District, Beijing, 100730 China; 2 Department of Nursing, Henan Provincial People’s Hospital, No.7 Weiwu Road, Jinshui District, Zhengzhou, 450003 China; 3 The Second Affiliated Hospital Zhejiang University School of Medicine, No. 88 Jiefang Road, Hangzhou, 310009 China; 4 Department of Nursing, Wuhan Union Hospital, No.1277 Jiefangdadao, Jianghan District, Wuhan, 430060 China; 5 Department of Nursing, Sichuan Provincial People’s Hospital, No. 32 West Second Section First Ring Road, Chengdu, 610072 China; 6 Department of Nursing, The First Affiliated Hospital, Sun Yat-sen University, No. 58 Zhongshan Second Road, Yuexiu District, Guangzhou, 200032 China

**Keywords:** Hospitalization costs, Stroke subtype, Prospective observational study, Multicenter, China

## Abstract

**Background:**

In this study, we aimed to analyze the hospitalization costs for immobile patients with hemorrhagic stroke (IHS) or ischemic stroke (IIS) in China and to determine the factors associated with hospitalization costs.

**Methods:**

We evaluated patients with IHS and IIS hospitalized between November 2015 and July 2016 in six provinces or municipality cities of China. Linear regression analysis was used to examine the association with hospitalization costs and predictors.

**Results:**

In total, 1573 patients with IHS and 3143 with IIS were enrolled and analyzed. For IHS and IIS, the average length of stay (LoS) was 17.40 ± 12.3 and 14.47 ± 11.55 days. The duration of immobility was 12.11 ± 9.98 and 7.36 ± 9.77 days, respectively. Median hospitalization costs were RMB 47000.68 (interquartile range 19,827.37, 91,877.09) for IHS and RMB 16578.44 (IQR 7020.13, 36,357.65) for IIS. In both IHS and IIS groups, medicine fees accounted for more than one-third of hospitalization costs. Materials fees and medical service fees accounted for the second and third largest proportions of hospital charges in both groups. Linear regression analysis showed that LoS, hospital level, and previous surgery were key determinants of hospitalization costs in all immobile patients with stroke. Subgroup analysis indicated that hospital level was highly correlated with hospitalization costs for IHS whereas pneumonia and deep vein thrombosis were key factors associated with hospitalization costs for IIS.

**Conclusions:**

We found that hospitalization costs were notably higher in IHS than IIS, and medicine fees accounted for the largest proportion of hospitalization costs in both patient groups, perhaps because most patients ended up with complications such as pneumonia thereby requiring more medications. LoS and hospital level may greatly affect hospitalization costs. Increasing the reimbursement ratio of medical insurance for patients with IHS is recommended. Decreasing medicine fees and LoS, preventing complications, and improving treatment capability may help to reduce the economic burden of stroke in China.

## Background

Stroke is a main cause of morbidity and mortality in low- and middle-income countries [1, 2]. China accounts for approximately one in three of the total deaths owing to stroke globally [3]. Estimates in 2013 were of nearly 2.4 million new strokes and 1.1 million stroke-related deaths, with 11.1 million stroke survivors alive at any given time [3, 4]. Stroke is also a serious disease in economic terms in China [5, 6]. The annual cost for stroke treatment was estimated to be RMB 37.5 billion in 2015, with the total cost rising to approximately RMB 50 billion if indirect costs are included [7].

With the lifestyle changes taking place in China together with economic growth, as well as an ongoing high prevalence, the number of patients with stroke and its hospitalization costs are rising [8, 9]. Although many medical care resources have been invested in stroke management [4], the costs for the main stroke subtypes vary owing to different potentially associated factors. Understanding the cost composition and the differences in stroke subtypes can help policymakers discern which types of cost and which contributors are driving increases in spending [10].

Previous studies have reported the economic burden of stroke treatment and care, but the results were limited to only one subtype of stroke [11, 12]. Retrospective observational studies have also been conducted, based on the Beijing Public Health Information Center database; however, this database only includes cost data from one region and therefore lacks representativeness [10–12]. Another study used cost data based on a single-center database, with a comparatively limited sample size [7]. In addition, previous reports have not focused on the hospitalization costs for immobile patients with stroke, who are often chronically impaired in their movement, often resulting in other comorbidities or medical complications that increase the economic burden to a certain extent [13, 14].

The present study was derived from a research project of the National Health and Family Planning Commission of the People’s Republic of China, which aimed to develop a standardized nursing intervention model (SNIM) among immobile patients with stroke. As part of baseline investigations for this large-scale prospective study, we carried out the present study, to provide a descriptive summary of factors associated with hospitalization cost estimates for immobile patients with hemorrhagic stroke (IHS) or ischemic stroke (IIS) in six different geographic regions of China. Furthermore, we explored differences in the contributors to ischemic and hemorrhagic stroke.

## Methods

### Study population

**Fig. 1:**
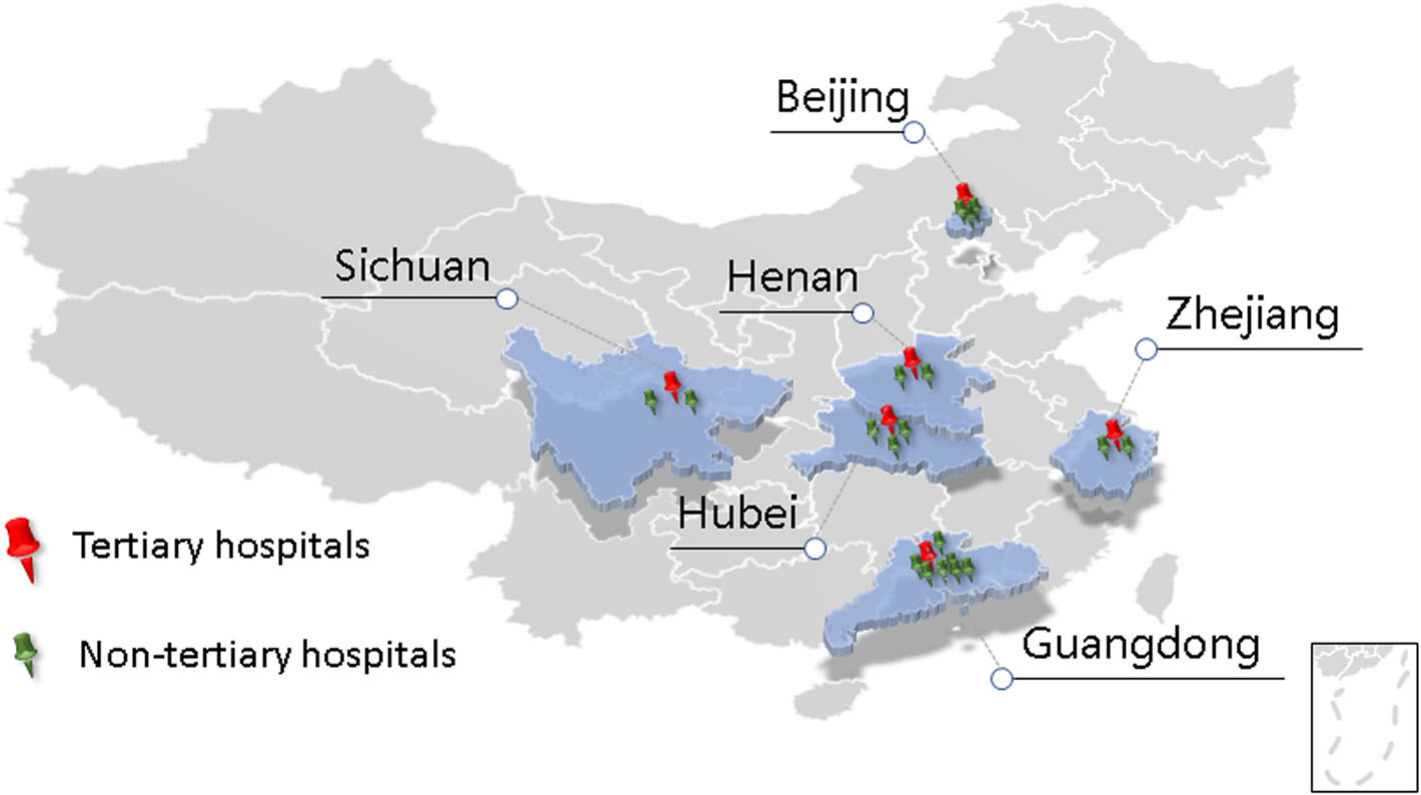
Location of hospitals included in this study. It was designed by the research team and edited by PowerPoint 2013. It was not taken from another source

Study participants were enrolled in the SNIM study, which was conducted in 25 public general hospitals covering six provinces or municipality cities of China (Beijing municipality city and Henan, Zhejiang, Guangdong, Hubei, and Sichuan provinces) from November 2015 to July 2016 (Fig. 1). Figure 1. was designed by the research team and edited by the PowerPoint 2013, it was not taken from another source. Further information about the SNIM study has been published elsewhere [15]. Patients with stroke comprised 4716 patients with principal diagnosis codes I60.x, I61.x, H34.1, I63.x, I64.x, according to the International Classification of Diseases, Tenth Revision. Patients were categorized according to hemorrhagic (I60.x, I61.x) and ischemic (H34.1, I63.x, I64.x) stroke [2, 16]. Immobility was defined as when the patient’s basic physiological needs were carried out in bed, except for active or passive bedside sitting/standing/wheelchair use for examination.

This study was ethically approved by the authorities of the 25 cooperating hospitals in six provinces or municipality cities of China, and participants signed a written informed consent before enrolment. All patient records and information were anonymized and deidentified prior to the analysis.

### Definition of covariates

Potential factors associated with hospitalization costs in the models included sociodemographic characteristics and clinical characteristics. Sociodemographic characteristics comprised age, sex, hospital level, and payment type. Clinical characteristics consisted of the patient’s body mass index (BMI); previous ICU admission; previous surgery; hospital length of stay (LoS); duration of immobility; major immobility complications (MICs) including pressure injury (PI), deep vein thrombosis (DVT), pneumonia, and urinary tract infection (UTI); invasive ventilation therapy; respiratory invasive operation; tracheotomy, urethral invasive operation; disorders of consciousness; immobility status at discharge; and Charlson Comorbidity Index (CCI) score. CCI was categorized according to scores of 0–3, 4, 5, and 6 or above. Total hospitalization costs consisted of costs for medicine, materials, medical services, laboratory and examination fees, nursing, blood transfusion, and other costs.

### Statistical analysis

Descriptive statistical methods were used for demographic characteristics. Continuous variables were described as mean ± standard deviation (SD) or median (interquartile range, IQR), and categorical variables were represented as number (percentage). In univariate analysis, we used the Wilcoxon signed-rank test and Kruskal–Wallis test to assess the associated factors. Then, associations of sociodemographic or clinical characteristics with hospitalization costs were analyzed using a multiple linear regression model. All parameters with a *P* < 0.05 in univariate analysis were included in the multivariate analyses. For categorical variables such as payment type, we used the “Enter” method, after transferring these to the dummy variables. Levels of significance *P* < 0.05 for inclusion and *P* > 0.10 for exclusion were used for the other parameters in the stepwise procedure. We used three multiple linear regression models for all immobile patients with stroke, immobile patients with hemorrhagic stroke (IHS), and immobile patients with ischemic stroke (IIS), respectively. A two-sided *P* value < 0.05 was considered to indicate statistical significance. IBM SPSS version 19 for Windows (IBM Corp, Armonk, NY, USA) was used for all statistical analyses.

## Results

### Demographics

We included a total 4716 immobile patients with stroke requiring hospitalization between November 2015 and July 2016 (Table 1). Mean patient age was 63.39 ± 14.48 years. Among the total, 1573 (33.35%) had hemorrhagic stroke, 3143 (66.64%) had ischemic stroke; 2726 (57.8%) were men and 1990 (42.2%) were women. In total, 36.7 and 35.3% of IHS had previous ICU admission and previous surgery, respectively; these rates were 12.7 and 12.1%, respectively, in IIS. The average LoS and duration of immobility was 15.45 ± 11.89 days and 8.94 ± 10.09 days, respectively, for all patients. IHS had longer LoS (17.40 ± 12.31 days vs. 14.47 ± 11.55 days) and duration of immobility (12.11 ± 9.98 days vs. 7.36 ± 9.77 days) than IIS. Medical insurance covered approximately 73% patients; 42.9% of patients were supported by the new cooperative medical system (NCMS) insurance whereas 20.0% were self-paying. A total 3158 (67.0%) patients were in tertiary hospitals and 1558 (33.0%) were in non-tertiary hospitals. The most common MIC among immobile patients with stroke was pneumonia. IHS received more medical treatment or nursing interventions than those with ischemic stroke, such as invasive ventilation therapy and respiratory or urethral invasive operations.

**Table 1:** Characteristics of immobile patients with stroke

	Total (*n* = 4716)	Hemorrhagic (*n* = 1573)	Ischemic (*n* = 3143)
Age group, n (%)			
18–44 years old	440(9.3%)	256(16.3%)	184(5.9%)
45–64 years old	1949(41.3%)	874(55.6%)	1075(34.2%)
65 years old and above	2327(49.3%)	443(28.2%)	1884(59.9%)
Sex, n (%)			
Male	2726(57.8%)	896(57.0%)	1830(58.2%)
Female	1990(42.2%)	677(43.0%)	1313(41.8%)
BMI	23.82 ± 3.34	23.89 ± 3.43	23.78 ± 3.30
Previous ICU admission, n (%)			
Yes	976(20.7%)	578(36.7%)	398(12.7%)
No	3740(79.3%)	995(63.3%)	2745(87.3%)
Previous surgery, n (%)			
Yes	936(19.8%)	556(35.3%)	380(12.1%)
No	3780(80.2%)	1017(64.7%)	2763(87.9%)
LoS	15.45 ± 11.89	17.40 ± 12.31	14.47 ± 11.55
Duration of immobility, n (%)	8.94 ± 10.09	12.11 ± 9.98	7.36 ± 9.77
Number of CCI, n (%)			
0–3	1471(31.2%)	861(54.7%)	610(19.4%)
4	973(20.6%)	389(24.7%)	584(18.6%)
5	1151(24.4%)	230(14.6%)	921(29.3%)
6 and above	1121(23.8%)	93(5.9%)	1028(32.7%)
Payment type, n (%)			
UEBMI	659(14.0%)	182(11.6%)	477(15.2%)
URBMI	759(16.1%)	179(11.4%)	580(18.5%)
NCMS	2023(42.9%)	636(40.4%)	1387(44.1%)
The Public Health Insurance Program	114(2.4%)	28(1.8%)	86(2.7%)
Self-paying	945(20.0%)	464(29.5%)	481(15.3%)
Others	216(4.6%)	84(5.3%)	132(4.2%)
Hospital level, n (%)			
Tertiary hospital	3158(67.0%)	1324(84.2%)	1834(58.4%)
Non-tertiary hospital	1558(33.0%)	249(15.8%)	1309(41.6%)
PI, n (%)			
Yes	60(1.3%)	26(1.7%)	34(1.1%)
No	4656(98.7%)	1547(98.3%)	3109(98.9%)
DVT, n (%)			
Yes	50(1.1%)	24(1.5%)	26(0.8%)
No	4666(98.9%)	1549(98.5%)	3117(99.2%)
Pneumonia, n (%)			
Yes	475(10.1%)	230(14.6%)	245(7.8%)
No	4241(89.9%)	1343(85.4%)	2898(92.2%)
UTI, n (%)			
Yes	64(1.4%)	36(2.3%)	28(0.9%)
No	4652(98.6%)	1537(97.7%)	3115(99.1%)
Invasive ventilation therapy, n (%)			
Yes	508(10.8%)	324(20.6%)	184(5.9%)
No	4208(89.2%)	1249(79.4%)	2959(94.1%)
Respiratory invasive operation			
Yes	60(1.3%)	40(2.5%)	20(0.6%)
No	4656(98.7%)	1533(97.5%)	3123(99.4%)
Tracheotomy			
Yes	641(13.6%)	413(26.3%)	228(7.3%)
No	4075(86.4%)	1160(73.7%)	2915(92.7%)
Urethral invasive operation			
Yes	1318(27.9%)	779(49.5%)	539(17.1%)
No	3398(72.1%)	794(50.5%)	2604(82.9%)
Disorder of consciousness, n (%)			
Yes	791(16.8%)	506(32.2%)	285(9.1%)
No	3925(83.2%)	1067(67.8%)	2858(90.9%)
Immobility status at discharge, n (%)			
Yes	1641(34.8%)	727(46.2%)	914(29.1%)
No	3075(65.2%)	846(53.8%)	2229(70.9%)

*Abbreviations*: *BMI* body mass index, *LoS* length of stay, *CCI* Charlson Comorbidity Index, *ICU* intensive care unit, *PI* pressure injury, *DVT* deep vein thrombosis, *UTI* urinary tract infection, *UEBMI* Urban Employee Basic Medical Insurance (to support employed workers), *URBMI* Urban Resident Basic Medical Insurance (to support urban residents without a stable job), *NCMS* new cooperative medical system (to support rural residents)

Notes: Public Health Insurance Program provides payments for retired officials who started work before 1949, civil servants, and government-affiliated employees. Self-paying means patients pay all hospital fees out of pocket. Others indicates other insurance such as private commercial insurance companies subsidizing basic insurance coverage

### Hospitalization costs among immobile patients with stroke

**Fig. 2:**
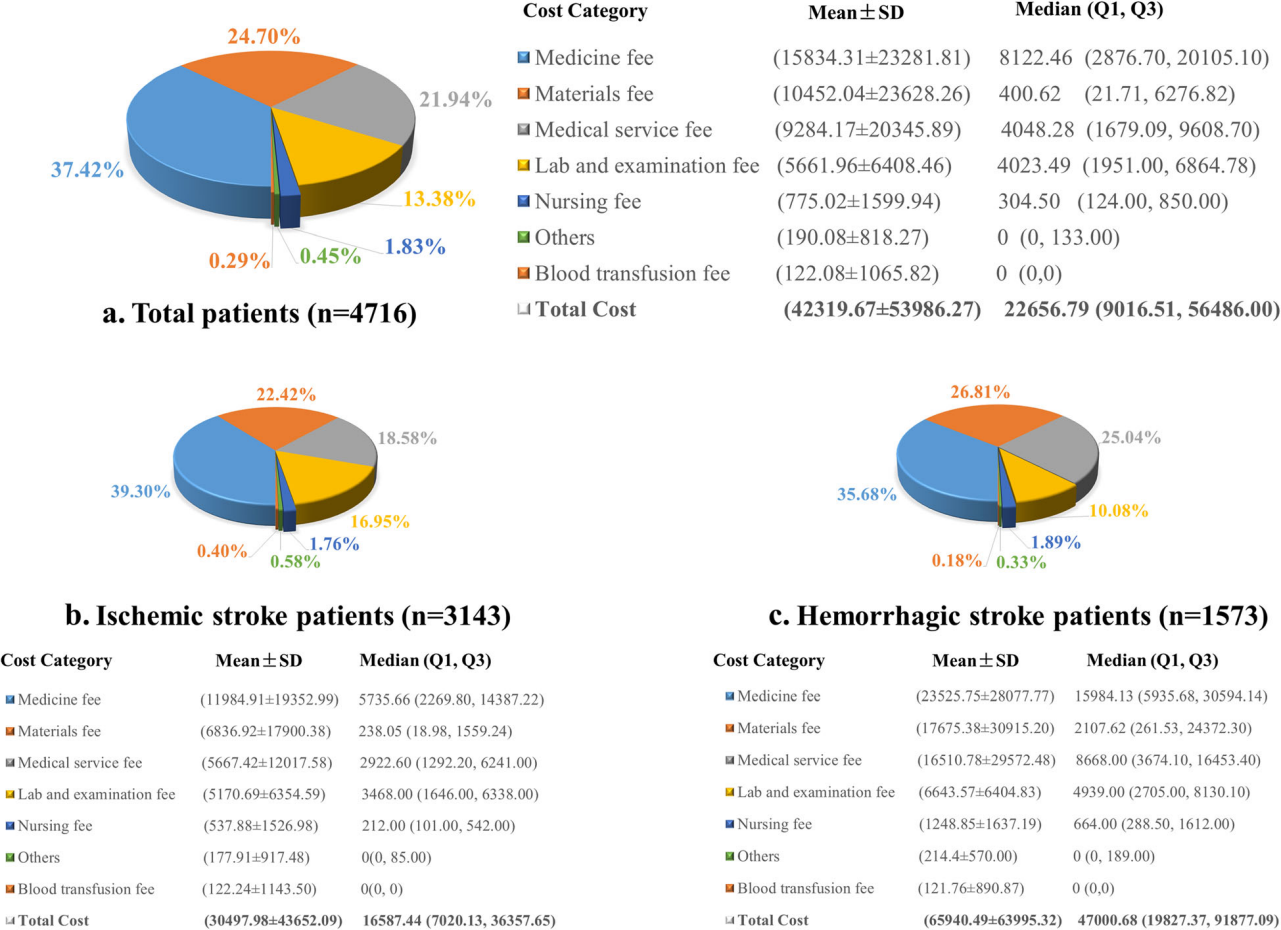
Composition ratio of hospitalization costs for immobile patients with stroke. Medicine fees include fees for Western medicine and traditional Chinese medicine. Medical service fees include diagnostic fees and fees for general treatment, surgical treatment, and other treatment. Laboratory and examination fees include fees for pathological examination, laboratory examination, and imaging examination. **a**. All patients with stroke (*n* = 4716). **b**. Patients with ischemic stroke (*n* = 3143). **c**. Patients with hemorrhagic stroke (*n* = 1573)

Figure 2 depicts hospitalization costs, according to category. The average hospitalization costs for all immobile patients with stroke was RMB 42,319.67 ± 53,986.27 and median hospitalization costs were RMB 22,656.79 (IQR RMB 9016.51, 56,486.00). Fees for medicine, materials, medical services, and laboratory and examination fees constituted 37.42, 24.70, 21.94, and 13.38% of the hospitalization costs in all immobile patients with stroke. Fees for nursing (1.83%), blood transfusion (0.29%), and other fees (0.45%) accounted for the minimum proportion in the distribution (Fig. 2a). In the subgroup analysis, the cost category distributions were consistent for the distribution of costs in IHS and IIS (Fig. 2b and c). In comparison with IIS, IHS had higher materials fees (26.81% vs. 22.42%), medical service fees (25.04% vs. 18.58%), and nursing fees (1.89% vs.1.76%).

### Potential factors associated with hospitalization costs

Supplementary Table 1 demonstrates the results of univariate analysis for the factors associated with hospitalization costs in all immobile patients with stroke. The findings suggest that stroke subtype, age, CCI score, previous ICU admission, previous surgery, payment type, hospital level, MICs, invasive ventilation therapy, respiratory invasive operation, tracheotomy, urethral invasive operation, disorders of consciousness, and immobility status at discharge were all associated with hospitalization costs.

Multivariate analysis was then carried out using those factors that were significant in the univariate analysis. Table 2 and Supplementary Table 4 show the results of multivariate analysis for hospitalization costs in immobile patients with stroke. In the stepwise regression model, LoS, hospital level, and previous surgery were found to be significant determinants of hospitalization costs (*P* < 0.001). The factors influencing coefficients of association, from highest to lowest, were LoS, hospital level, previous surgery, previous ICU admission, invasive ventilation therapy, urethral invasive operation, respiratory invasive operation, pneumonia, and stroke subtype (adjusted R^2^ = 0.512, *P* < 0.001).

**Table 2:** Multivariate analysis for factors associated with hospitalization costs for immobile patients with stroke (adjusted R^2^ = 0.512)

	regression coefficient		standardization coefficient	***t***	***p***
	B	SE			
constant	−15,425.23	1185.55		−13.01	< 0.001
LoS	1435.76	48.53	0.316	29.585	< 0.001
Hospital level	25,918.73	1270.08	0.226	20.407	< 0.001
Previous surgery	25,707.62	1573.24	0.190	16.341	< 0.001
Previous ICU admission	18,932.86	1631.18	0.142	11.607	< 0.001
Urethral invasive operation	11,917.18	1342.77	0.106	8.875	< 0.001
Invasive ventilation therapy	20,361.28	2193.23	0.117	9.284	< 0.001
Respiratory invasive operation	36,370.48	4474.34	0.084	8.129	< 0.001
Pneumonia	11,114.14	1990.73	0.062	5.583	< 0.001
Stroke subtype (hemorrhagic stroke)	5660.20	1286.94	0.049	4.398	< 0.001

*Abbreviation*: *LoS* length of stay, *ICU* intensive care unit, *SE* standard error

### Subgroup analysis of the factors associated with hospitalization costs

The results of univariate analysis for factors associated with hospitalization costs in IHS and IIS are shown in Supplementary Tables 2 and 3. Multivariate analysis was performed using the factors that were significant in the univariate analysis. The statistically significant predictors of hospitalization costs of IHS and IIS in the stepwise multiple linear regression model are shown in Tables 3 and 4 and Supplementary Table 4. According to standardized coefficients, the associated factors in IHS, in decreasing order, were hospital level, LoS, previous surgery, duration of immobility, previous ICU admission, respiratory invasive operation, urethral invasive operation, and invasive ventilation therapy (adjusted R^2^ = 0.421, *P* < 0.001). In IIS (see Table 4), the factors associated with hospitalization costs, in decreasing order, were LoS, hospital level, previous ICU admission, previous surgery, invasive ventilation therapy, urethral invasive operation, respiratory invasive operation, pneumonia, and DVT (adjusted R^2^ = 0.536, *P* < 0.001).

**Table 3:** Multivariate analysis for factors associated with hospitalization costs in immobile patients with hemorrhagic stroke (adjusted R^2^ = 0.421)

	regression coefficient		standardization coefficient	***t***	***p***
	B	SE			
constant	−32,229.38	3939.29		−8.182	< 0.001
Hospital level	45,490.24	3630.41	0.260	12.53	< 0.001
LoS	1183.95	147.76	0.228	8.013	< 0.001
Previous surgery	30,524.34	2798.87	0.228	10.906	< 0.001
Duration of immobility	930.61	186.11	0.145	5	< 0.001
Previous ICU admission	18,562.96	3051.55	0.140	6.083	< 0.001
Respiratory invasive operation	38,566.54	7442.69	0.102	5.182	< 0.001
Urethral invasive operation	12,211.79	2568.11	0.101	4.755	< 0.001
Invasive ventilation therapy	15,288.83	3670.33	0.097	4.166	0.001

*Abbreviations*: *LoS* length of stay, *ICU* intensive care unit, *SE* standard error

**Table 4:** Multivariate analysis for factors associated with hospitalization costs for immobile patients with ischemic stroke (adjusted R^2^ = 0.536)

	regression coefficient		standardization coefficient	***t***	***p***
	B	SE			
constant	−11,583.29	1029.58		−11.251	< 0.001
LoS	1391.41	48.35	0.368	28.779	< 0.001
Hospital level	20,851.95	1145.30	0.236	18.207	< 0.001
Previous ICU admission	20,012.31	1815.94	0.152	11.020	< 0.001
Invasive ventilation therapy	27,413.61	2665.64	0.147	10.284	< 0.001
Previous surgery	19,936.57	1833.14	0.149	10.876	< 0.001
Urethral invasive operation	10,338.59	1454.75	0.099	7.107	< 0.001
DVT	30,242.50	5954.05	0.063	5.079	< 0.001
Respiratory invasive operation	30,398.73	5639.84	0.067	5.390	< 0.001
Pneumonia	10,960.55	2085.26	0.067	5.256	< 0.001

*Abbreviations*: *LoS* length of stay, *ICU* intensive care unit, *SE* standard error, *DVT* deep vein thrombosis

## Discussion

In China, the stroke burden is expected to increase further as a result of population aging and an ongoing high prevalence of risk factors for stroke; however, the costs for different stroke subtypes have not been comprehensively analyzed. Describing the distribution and characteristics of the hospitalization costs in stroke and examining the associated factors are essential steps to providing high-quality health care services while avoiding increasing the socioeconomic burden. To the best of our knowledge, our study is the latest study that analyzes the distribution and potential factors associated with hospitalization costs for different subtypes of stroke in the Chinese population, based on a prospective multicenter study.

In the present study, the mean (median) hospitalization costs per person were RMB 442,319.67 ± 53,986.27 (RMB 22,656.79; IQR 9016.51, 56,486.00), which are lower than those in developed countries of North America and Europe, as well as in Japan. A study in the United States indicated that the average cost of stroke was USD 20,396 ± 23,256 (RMB 141,652.26 ± 161,515.25, the spending estimates were converted to RMB (CNY) based on exchange rates in 2008 (1 USD = 6.9451 CNY)) [17]. Research from Germany suggested that the mean hospitalization cost for hemorrhagic stroke was 26,602 USD (RMB 220,222.00, the spending estimates were converted to RMB (CNY) based on exchange rates in 2000 (1 USD = 8.2784 CNY)) [18]. Tu et al. studied hospitalized patients with ischemic stroke in Japan (from 1995 to 1999) and found that the mean (median) hospital charges per patient were USD 9020 (USD 7974) (RMB 74,681.99 (RMB 66,021.53), the spending estimates were converted to RMB (CNY) based on exchange rates in 1999 (1 USD = 8.2796 CNY)) [19].

Additionally, hospitalization costs were notably higher in IHS than IIS (RMB 47,000.68, IQR 19,827.37, 91,877.09 vs. RMB 16,587.44, IQR 7020.13, 36,357.65). It is plausible that IHS tend to require more complex medical treatment or nursing interventions than IIS; thus, the median materials fees, medical service fees, and total costs for patients with severe illness may be higher in IHS. This is in line with the results of other resource-use studies indicating higher costs in patients with hemorrhagic stroke than in those who have ischemic stroke [10, 11]. Therefore, it is necessary to increase the reimbursement ratio of medical insurance for patients with hemorrhagic stroke, to reduce the personal medical expenses of these patients.

As for cost composition analysis, we found that medicine fees represented the largest proportion of overall hospitalization costs (as high as 37.42%); materials fees (24.70%) and medical service fees (21.94%) ranked second and third in the total hospitalization costs. Also, the composition of hospitalization costs is parallel in IHS or IIS. Compared with other studies carried out in China, the cost composition is mostly consistent with those in previous studies whereas the proportion of medicine fees is lower [7, 10, 11]. This may be attributable to the large reform to China’s health system implemented in public hospitals during the past decade, which imposed zero markup on drug costs and encouraged the use of inexpensive medications [20, 21].

In contrast, the cost composition in our study was quite different from those reported in Western countries. In Greece, Gioldasis et al. indicated that only about 7% of the total charges for stroke was attributable to medicines [22]. In Germany, Dodel et al. suggested that the costs for medicines were low, with a mean total cost of EUR 120 ± 240 [18]. Asil et al. analyzed the direct costs of acute ischemic and hemorrhagic stroke in Turkey, indicating that 29.9% of the total charge was for medicine [23]. For one thing, perhaps because most patients enrolled in this study ended up with complications thereby requiring more medications. For another thing, the Chinese government has made great efforts to reduce prescribing and medicine costs; however, the government must further standardize medical treatment and introduce additional and more effective policies. Dodel et al. suggested that nursing fees are related to the hospital LoS [18]. The average LoS in this study was 15.45 ± 11.89 days for all patients with stroke, which is longer than in developed countries, yet the nursing fees only accounted for a minimum proportion (1.83%) of the total costs, which is lower than the 8% in Greece [22]. However, countries may differ in their developmental level and medical insurance service systems. Therefore, longitudinal comparisons within countries in this regard are more important than comparisons between countries.

In linear regression analysis, LoS, hospital level, previous surgery, previous ICU admission, invasive ventilation therapy, urethral invasive operation, respiratory invasive operation, pneumonia, and stroke subtype (hemorrhagic stroke) were found to be significantly associated with hospitalization costs for all immobile patients with stroke in this study. Identifying such factors can help to understand the nature of hospitalization expenses, so as to improve the efficiency of health care delivery. LoS was highly correlated with the hospitalization costs for immobile patients with stroke, which is in accordance with the results of several previous studies [11, 12, 17, 22, 23]. LoS is closely related to medical complications after stroke [24], the complication rates in this study were higher than those for previous studies; thus, as a controllable factor, LoS can be shortened by preventing nosocomial infections and medical complications. Other contributors such as hospital level, previous surgery, previous ICU admission, invasive ventilation therapy, urethral and respiratory invasive operations, and stroke subtype may represent the severity of illness.

The average LoS for all immobile patients with stroke was 15.45 ± 11.89 days in this study, which is longer than in previous reports from China [10, 11] and Europe [18, 22]. In most countries, patients with stroke are hospitalized for a short period of 10 to 15 days [5]. The potential reasons for the difference in our results may be that patients in this study were immobile and were therefore more susceptible to medical complications such as pneumonia, PI, and so on, which could prolong LoS [24, 25]. Moreover, patients with hemorrhagic stroke had longer LoS than those with ischemic stroke, which was consistent with earlier reports on ischemic and hemorrhagic stroke in China from 2015 to 2018 [10–12]. Previous studies have identified that payment type influences stroke costs [6, 10]. In our study, 2023 patients (42.9%) who were covered by the NCMS had lower costs, possibly because this type of payment forces hospitals to control costs with shorter LoS.

Subgroup analysis indicated that hospital level was highly correlated with the hospitalization costs for IHS. The potential reasons for this difference may be that hemorrhagic stroke is less prevalent but more likely to be fatal [26], and patients with more severe physical impairment definitely require more medical care resources [12, 15, 27]. Tertiary hospitals are top-level hospitals in China, in which the health services and medical resources are more advanced. Therefore, patients with severe neurological impairment tend to be treated in tertiary hospitals. Furthermore, the professional level of physicians in tertiary hospitals is generally higher, and more advanced professional skills are related to higher medical service fees [11, 12, 22].

Our study findings also suggested that pneumonia and DVT were significantly associated with hospitalization costs in IIS. However, the complication rates in our study were somewhat higher than those of previous studies (DVT: 0.8% vs. 0.2% [28]; pneumonia: 7.8% vs. 4.6% [29]). Balami et al. [30] and Bustamante et al. [31] indicated that complications after ischemic stroke could prolong the LoS, which may explain the impact of DVT and pneumonia on hospitalization costs for ischemic stroke [24, 32].

The duration of immobility was another significant predictor in the patient subgroup with hemorrhagic but not ischemic stroke. The duration of immobility after hemorrhagic stroke was longer than that after ischemic stroke (12.11 ± 9.98 days vs. 7.36 ± 9.77 days) in our sample. In addition, complications such as pneumonia or DVT can arise as a direct consequence of stroke itself, owing to ensuing immobility or disability [24]. These complications present barriers to optimal recovery and positive clinical outcomes or they can increase the costs of hospital care when complications are non-fatal.

This study has some limitations. The cost data reported in the present study reflect only direct health care spending and do not account for indirect societal costs associated with rehabilitation and intangible costs to patients with stroke; further research is required to estimate these costs and to obtain an estimate of the total costs for immobile patients with stroke. Given the cross-sectional nature of this study, we could not determine causation or the direction of the observed relationships. In addition, we were unable to assess all potentially associated factors that may determine hospitalization costs, such as alcohol use. For the univariate and multivariate analysis, when there is a large sample size, there is a tendency for small differences to become statistically significant. In Supplementary Tables 1 and 2, we can find actual cost difference is also very obvious in different categorical variables layers (such as subtype of stroke, age group, experience of surgery, experience of ICU, hospital level). This made us think that the test power of the analysis was in a relatively reasonable range. However, a more rigorous analytical method with more sophisticated evaluations are required to confirm our findings. The etiology, complications, and comorbidities for an ischemic stroke may potentially influence the initial cost of hospitalization. We will continue to explore in depth in the next step of the study.

The strength of our study is that we analyzed the distribution and predictors associated with hospitalization costs for different subtypes of stroke; patients enrolled in this study were from six regions of China, and this work was based on a prospective multicenter study. Therefore, our results will be valuable in economic evaluations to support policymaking regarding reimbursement, investment, and pricing for medical or nursing interventions. This study is of practical utility in developing countries and some developed countries aiming to provide comprehensive health services in immobile patients with stroke, to better balance patients’ health gains, personal costs, and social welfare costs.

## Conclusion

The findings of our study suggest that the cost composition for immobile patients with stroke in China is quite different from that in Western countries. The hospitalization costs are notably higher in IHS than in IIS. Medicine fees accounted for the largest proportion of hospitalization costs for these patients, far outweighing the proportions in developed countries of North America and Europe, perhaps because most patients ended up with complications such as pneumonia thereby requiring more medications. In addition, we found that LoS and hospital level were critical factors associated with hospitalization costs in immobile patients with both hemorrhagic and ischemic stroke. Reducing the economic burden of stroke in China can most likely be achieved by increasing the reimbursement ratio of medical insurance for IHS, decreasing medicine fees and LoS, preventing complications, and improving treatment capabilities.

## Supplementary information

**Additional file 1: Table S1.** Univariate analysis for factors associated with hospitalization costs in all patients with stroke (*n* = 4716). 1) Wilcoxon signed-rank test; 2) Kruskal–Wallis test. Abbreviations: Q1, 1st quartile (25%); Q3, 3rd quartile (75%); CCI, Charlson Comorbidity Index; ICU, intensive care unit; PI, pressure injury; DVT, deep vein thrombosis; UTI, urinary tract infection; UEBMI, Urban Employee Basic Medical Insurance; URBMI, Urban Resident Basic Medical Insurance; NCMS, New Cooperative Medical System. **Table S2.** Univariate analysis for factors associated with hospitalization costs in patients with hemorrhagic stroke (*n* = 1573). 1) Wilcoxon signed-rank test; 2) Kruskal–Wallis test. Abbreviations: Q1, 1st quartile (25%); Q3, 3rd quartile (75%); CCI, Charlson Comorbidity Index; ICU, intensive care unit; PI, pressure injury; DVT, deep vein thrombosis; UTI, urinary tract infection; UEBMI, Urban Employee Basic Medical Insurance; URBMI, Urban Resident Basic Medical Insurance; NCMS, New Cooperative Medical System. **Table S3.** Univariate analysis for factors associated with hospitalization costs in patients with ischemic stroke (*n* = 3143). 1) Wilcoxon signed-rank test; 2) Kruskal–Wallis test. Abbreviations: Q1, 1st quartile (25%); Q3, 3rd quartile (75%); CCI, Charlson Comorbidity Index; ICU, intensive care unit; PI, pressure injury; DVT, deep vein thrombosis; UTI, urinary tract infection; UEBMI, Urban Employee Basic Medical Insurance; URBMI, Urban Resident Basic Medical Insurance; NCMS, New Cooperative Medical System. **Table S4.** Multivariate analysis for factors associated with hospitalization costs for immobile patients with stroke. 1) Adjusted R^2^ = 0.512; 2) adjusted R^2^ = 0.536; 3) adjusted R^2^ = 0.421. * *P* < 0.001. Abbreviations: LoS, length of stay; ICU, intensive care unit; SE, standard error; DVT, deep vein thrombosis.

## Data Availability

The datasets generated for this study are available on request to the corresponding author.

## Abbreviations

BMI: Body mass index
LoS: Length of stay
CCI: Charlson Comorbidity Index
ICU: Intensive care unit
PI: Pressure injury
DVT: Deep vein thrombosis
UTI: Urinary tract infection
UEBMI: Urban Employee Basic Medical Insurance (to support employed workers)
URBMI: Urban Resident Basic Medical Insurance (to support urban residents without a stable job)
NCMS: New cooperative medical system (to support rural residents)
Q1: 1st quartile (25%)
Q3: 3rd quartile (75%)
SE: Standard error
SNIM: Standardized nursing intervention model
