# Supplementary material for: Hospitalization costs among immobile patients with hemorrhagic or ischemic stroke in China: a multicenter cross-sectional study
Source: BMC Health Serv Res. 2020 Sep 29;20:905. doi: 10.1186/s12913-020-05758-6 (PMC7526192; doi:10.1186/s12913-020-05758-6)
Supplement: Supplementary file 1 — Additional file 1: Table S1. Univariate analysis for factors associated with hospitalization costs in all patients with stroke (n = 4716). 1) Wilcoxon signed-rank test; 2) Kruskal–Wallis test. Abbreviations: Q1, 1st quartile (25%); Q3, 3rd quartile (75%); CCI, Charlson Comorbidity Index; ICU, intensive care unit; PI, pressure injury; DVT, deep vein thrombosis; UTI, urinary tract infection; UEBMI, Urban Employee Basic Medical Insurance; URBMI, Urban Resident Basic Medical Insurance; NCMS, New Cooperative Medical System. Table S2. Univariate analysis for factors associated with hospitalization costs in patients with hemorrhagic stroke (n = 1573). 1) Wilcoxon signed-rank test; 2) Kruskal–Wallis test. Abbreviations: Q1, 1st quartile (25%); Q3, 3rd quartile (75%); CCI, Charlson Comorbidity Index; ICU, intensive care unit; PI, pressure injury; DVT, deep vein thrombosis; UTI, urinary tract infection; UEBMI, Urban Employee Basic Medical Insurance; URBMI, Urban Resident Basic Medical Insurance; NCMS, New Cooperative Medical System. Table S3. Univariate analysis for factors associated with hospitalization costs in patients with ischemic stroke (n = 3143). 1) Wilcoxon signed-rank test; 2) Kruskal–Wallis test. Abbreviations: Q1, 1st quartile (25%); Q3, 3rd quartile (75%); CCI, Charlson Comorbidity Index; ICU, intensive care unit; PI, pressure injury; DVT, deep vein thrombosis; UTI, urinary tract infection; UEBMI, Urban Employee Basic Medical Insurance; URBMI, Urban Resident Basic Medical Insurance; NCMS, New Cooperative Medical System. Table S4. Multivariate analysis for factors associated with hospitalization costs for immobile patients with stroke. 1) Adjusted R2 = 0.512; 2) adjusted R2 = 0.536; 3) adjusted R2 = 0.421. * P < 0.001. Abbreviations: LoS, length of stay; ICU, intensive care unit; SE, standard error; DVT, deep vein thrombosis. [file 12913_2020_5758_MOESM1_ESM.docx]

**Supplementary Table 1. Univariate analysis for factors associated with hospitalization costs in all patients with** **stroke (n=4716).**

|  | **Cost**  **Median (Q1, Q3)** | **Z/H** | ***P* value** |
| --- | --- | --- | --- |
| Subtype of stroke |  | -25.152^1)^ | <0.001 |
| Hemorrhagic stroke | 47000.68(19827.37, 91877.09) |  |  |
| Ischemic stroke | 16587.44(7020.13, 36357.65) |  |  |
| Age |  | 141.720^2)^ | <0.001 |
| 18-44 years old | 37889.07(17538.57, 75115.23) |  |  |
| 45-64 years old | 26401.14(10732.48, 63880.21) |  |  |
| 65 years old and above | 18118.60(7516.99, 43692.93) |  |  |
| Sex |  | -1.056^1)^ | 0.291 |
| Male | 23551.95(9689.63, 55042.08) |  |  |
| Female | 21957.76(8269.24, 58498.13) |  |  |
| Number of CCI |  | 215.011^2)^ | <0.001 |
| 0-3 | 35184.17(14882.95, 72906.79) |  |  |
| 4 | 22310.95(8743.17, 58442.19) |  |  |
| 5 | 17206.25(7061.81, 38582.33) |  |  |
| 6 and above | 17691.65(8115.59, 40428.70) |  |  |
| Previous ICU admission |  | -27.225^1)^ | <0.001 |
| Yes | 64763.03(29625.17, 114648.07) |  |  |
| No | 17723.43(7608.51, 39441.29) |  |  |
| Previous surgery |  | -33.074^1)^ | <0.001 |
| Yes | 75771.63(47783.17, 112587.27) |  |  |
| No | 17153.98(7611.02, 34895.35) |  |  |
| Payment type |  | 281.465^2)^ | <0.001 |
| UEBMI | 22098.52(8205.45, 58637.21) |  |  |
| URBMI | 27507.41(13821.85, 57983.30) |  |  |
| NCMS | 14896.80(6137.20, 41805.44) |  |  |
| The Public Health Insurance Program | 30101.49(16904.79, 62684.07) |  |  |
| Self-paying | 29769.22(13372.82, 67969.60) |  |  |
| Others | 45032.29(20002.17, 83142.85) |  |  |
| Hospital level |  | -40.585^1)^ | <0.001 |
| Tertiary hospital | 37147.44(18567.30, 74970.23) |  |  |
| Non-tertiary hospital | 7414.31(4424.41, 14102.41) |  |  |
| PI |  | -7.405^1)^ | <0.001 |
| Yes | 75694.43(35939.83, 178467.63) |  |  |
| No | 22231.74(8899.74, 55794.17) |  |  |
| DVT |  | -6.992^1)^ | <0.001 |
| Yes | 63142.70(44484.06, 151325.88) |  |  |
| No | 22274.38(8912.33, 55881.22) |  |  |
| Pneumonia |  | -16.361^1)^ | <0.001 |
| Yes | 63946.50(26577.63, 118242.57) |  |  |
| No | 20534.88(8302.70, 49699.26) |  |  |
| UTI |  | -3.499^1)^ | <0.001 |
| Yes | 40114.80(21023.59, 93856.72) |  |  |
| No | 22370.82(8923.02, 56166.56) |  |  |
| Invasive ventilation therapy |  | -25.726^1)^ | <0.001 |
| Yes | 90193.39(55826.67, 142713.17) |  |  |
| No | 19545.35(8190.70, 43980.02) |  |  |
| Respiratory invasive operation |  | -8.129^1)^ | <0.001 |
| Yes | 98111.64(56183.74, 179618.44) |  |  |
| No | 22207.03(8891.15, 55113.59) |  |  |
| Tracheotomy |  | -26.731^1)^ | <0.001 |
| Yes | 76380.68(47930.59, 117305.74) |  |  |
| No | 18651.26(8018.15, 40654.69) |  |  |
| Urethral invasive operation |  | -32.814^1)^ | <0.001 |
| Yes | 64328.29(33140.60, 103728.44) |  |  |
| No | 15811.43(7205.82, 32084.19) |  |  |
| Disorder of consciousness |  | -23.192^1)^ | <0.001 |
| Yes | 61491.08(30045.01, 98254.82) |  |  |
| No | 18527.17(7793.18, 42914.55) |  |  |
| Immobility status at discharge |  | -15.181^1)^ | <0.001 |
| Yes | 33336.76(15386.91, 69777.20) |  |  |
| No | 17978.44(7257.24, 45201.96) |  |  |

1) Wilcoxon rank test; 2) Kruskal–Wallis test.

Abbreviations: Q1, 1st quartile (25%); Q3, 3rd quartile (75%); CCI, Charlson Comorbidity Index; ICU, intensive care unit; PI, pressure injury; DVT, deep vein thrombosis; UTI, urinary tract infection; UEBMI, Urban Employee Basic Medical Insurance; URBMI, Urban Resident Basic Medical Insurance; NCMS, New Cooperative Medical System.

**Supplementary Table 2. Univariate analysis for factors associated with hospitalization costs in patients with** **hemorrhagic stroke (n=1573).**

|  | **Cost**  **Median (Q1, Q3)** | **Z/H** | ***P* value** |
| --- | --- | --- | --- |
| Age |  | 11.615^2)^ | 0.003 |
| 18-44 years old | 54518.66(23861.19, 90147.68) |  |  |
| 45-64 years old | 52211.63(21074.66, 96866.24) |  |  |
| 65 years old and above | 37016.27(15865.63, 84087.01) |  |  |
| Sex |  | -3.183^1)^ | 0.001 |
| Male | 42484.41(18469.38, 84064.83) |  |  |
| Female | 56064.67(22189.48, 99713.05) |  |  |
| Number of CCI |  | 17.870^2)^ | <0.001 |
| 0-3 | 52386.82(22339.15, 95664.74) |  |  |
| 4 | 49353.75(19274.96, 92319.67) |  |  |
| 5 | 37236.98(15752.94, 77563.22) |  |  |
| 6 and above | 26242.12(13967.30, 75668.10) |  |  |
| Previous ICU admission |  | -13.024^1)^ | <0.001 |
| Yes | 74686.31(34968.30, 123874.49) |  |  |
| No | 34052.97(15446.79, 69023.15) |  |  |
| Previous surgery |  | -18.683^1)^ | <0.001 |
| Yes | 85490.58(56800.14, 121637.39) |  |  |
| No | 29183.75(14171.02, 60927.04) |  |  |
| Payment type |  | 12.873^2)^ | 0.025 |
| UEBMI | 44302.32(18600.26, 113243.95) |  |  |
| URBMI | 56235.58(24013.32, 105945.91) |  |  |
| NCMS | 45890.99(18798.68, 87470.56) |  |  |
| The Public Health Insurance Program | 31087.98(15753.56, 69920.88) |  |  |
| Self-paying | 44257.03(19716.63, 83055.91) |  |  |
| Others | 58390.13(21207.62, 122568.61) |  |  |
| Hospital level |  | -13.903^1)^ | <0.001 |
| Tertiary hospital | 56253.39(25099.44, 100313.34) |  |  |
| Non-tertiary hospital | 16019.91(8726.02, 30848.84) |  |  |
| PI |  | -4.858^1)^ | <0.001 |
| Yes | 121794.91(70820.13, 202750.38) |  |  |
| No | 45766.63(19363.47, 89950.21) |  |  |
| DVT |  | -2.424^1)^ | 0.015 |
| Yes | 72512.05(45693.67, 130026.81) |  |  |
| No | 46287.65(19428.51, 90645.72) |  |  |
| Pneumonia |  | -10.326^1)^ | <0.001 |
| Yes | 91603.22(50000.98, 138226.81) |  |  |
| No | 40025.59(17827.97, 81983.52) |  |  |
| UTI |  | -1.337^1)^ | 0.181 |
| Yes | 57096.50(27421.81, 108317.74) |  |  |
| No | 46582.01(19428.51, 91362.76) |  |  |
| Invasive ventilation therapy |  | -14.652^1)^ | <0.001 |
| Yes | 93771.40(59848.43, 142710.97) |  |  |
| No | 36054.49(16238.36, 74041.64) |  |  |
| Respiratory invasive operation |  | -5.526^1)^ | <0.001 |
| Yes | 113311.65(64571.05, 213741.43) |  |  |
| No | 45201.96(19225.24, 89776.10) |  |  |
| Tracheotomy |  | -14.247^1)^ | <0.001 |
| Yes | 80890.80(53754.87, 121039.93) |  |  |
| No | 32883.12(15653.76, 72797.31) |  |  |
| Urethral invasive operation |  | -14.879^1)^ | <0.001 |
| Yes | 69775.05(37049.36, 112399.19) |  |  |
| No | 27984.30(13465.52, 61482.28) |  |  |
| Disorder of consciousness |  | -9.331^1)^ | <0.001 |
| Yes | 64723.38(33901.19, 109559.50) |  |  |
| No | 35794.46(15727.48, 82586.48) |  |  |
| Immobility status at discharge |  | -1.356^1)^ | 0.175 |
| Yes | 49041.18(20788.74, 95376.83) |  |  |
| No | 44732.30(18955.34, 88426.51) |  |  |

1) Wilcoxon rank test; 2) Kruskal–Wallis test.

Abbreviations: Q1, 1st quartile (25%); Q3, 3rd quartile (75%); CCI, Charlson Comorbidity Index; ICU, intensive care unit; PI, pressure injury; DVT, deep vein thrombosis; UTI, urinary tract infection; UEBMI, Urban Employee Basic Medical Insurance; URBMI, Urban Resident Basic Medical Insurance; NCMS, New Cooperative Medical System.

**Supplementary Table 3. Univariate analysis for factors associated with hospitalization costs in patients with** **ischemic stroke (n=3143).**

|  | **Cost**  **Median (Q1, Q3)** | **Z/H** | ***P* value** |
| --- | --- | --- | --- |
| Age |  | 22.033^2)^ | <0.001 |
| 18-44 years old | 26141.55(11744.17, 49590.49) |  |  |
| 45-64 years old | 16903.66(7287.56, 35949.86) |  |  |
| 65 years old and above | 15390.00(6528.90, 35253.91) |  |  |
| Sex |  | -3.815^1)^ | <0.001 |
| Male | 17658.96(7552.20, 37839.43) |  |  |
| Female | 14754.39(6304.58, 33681.30) |  |  |
| Number of CCI |  | 38.490^2)^ | <0.001 |
| 0-3 | 21852.14(8780.55, 41892.03) |  |  |
| 4 | 13710.33(6074.05, 32660.12) |  |  |
| 5 | 13756.63(5979.66, 30921.10) |  |  |
| 6 and above | 17166.05(7682.21, 37238.71) |  |  |
| Previous ICU admission |  | -18.938^1)^ | <0.001 |
| Yes | 52239.95(24786.83, 95516.47) |  |  |
| No | 13901.18(6274.81, 29874.66) |  |  |
| Previous surgery |  | -22.371^1)^ | <0.001 |
| Yes | 64282.52(40712.80, 94622.00) |  |  |
| No | 13563.37(6317.28, 28019.76) |  |  |
| Payment type |  | 378.341^2)^ | <0.001 |
| UEBMI | 18346.70(6652.30, 39208.38) |  |  |
| URBMI | 22111.48(12123.58, 46607.06) |  |  |
| NCMS | 9297.64(4815.40, 24215.52) |  |  |
| The Public Health Insurance Program | 29874.66(17237.81, 54606.08) |  |  |
| Self-paying | 23483.95(11401.82, 51266.52) |  |  |
| Others | 39996.68(18387.87, 74980.68) |  |  |
| Hospital level |  | -35.576^1)^ | <0.001 |
| Tertiary hospital | 29019.77(16602.28, 57824.82) |  |  |
| Non-tertiary hospital | 6666.34(4119.91, 11384.76) |  |  |
| PI |  | -5.675^1)^ | <0.001 |
| Yes | 45600.30(31395.11, 126052.10) |  |  |
| No | 16399.88(6935.02, 35936.70) |  |  |
| DVT |  | -6.184^1)^ | <0.001 |
| Yes | 61196.10(42494.45, 164016.45) |  |  |
| No | 16399.88(6945.37, 35881.39) |  |  |
| Pneumonia |  | -10.722^1)^ | <0.001 |
| Yes | 33985.84(17186.57, 84213.40) |  |  |
| No | 15211.77(6596.92, 33580.15) |  |  |
| UTI |  | -1.841^1)^ | 0.066 |
| Yes | 28254.22(10323.26, 59744.95) |  |  |
| No | 16515.78(6966.23, 36256.24) |  |  |
| Invasive ventilation therapy |  | -17.011^1)^ | <0.001 |
| Yes | 80502.97(44735.00, 142748.03) |  |  |
| No | 15060.42(6614.07, 31653.66) |  |  |
| Respiratory invasive operation |  | -4.031^1)^ | <0.001 |
| Yes | 90923.57(39005.01, 129986.77) |  |  |
| No | 16468.61(6966.23, 35992.46) |  |  |
| Tracheotomy |  | -17.493^1)^ | <0.001 |
| Yes | 69464.22(42028.64, 109488.33) |  |  |
| No | 14798.84(6570.95, 30783.87) |  |  |
| Urethral invasive operation |  | -23.206^1)^ | <0.001 |
| Yes | 56770.77(30059.59, 88001.53) |  |  |
| No | 12877.39(6073.49, 26792.35) |  |  |
| Disorder of consciousness |  | -15.350^1)^ | <0.001 |
| Yes | 50579.49(25387.50, 81493.64) |  |  |
| No | 14742.85(6437.76, 31413.91) |  |  |
| Immobility status at discharge |  | -13.790^1)^ | <0.001 |
| Yes | 26018.78(12997.89, 52721.12) |  |  |
| No | 12564.38(5820.62, 29684.73) |  |  |

1) Wilcoxon rank test; 2) Kruskal–Wallis test.

Abbreviations: Q1, 1st quartile (25%); Q3, 3rd quartile (75%); CCI, Charlson Comorbidity Index; ICU, intensive care unit; PI, pressure injury; DVT, deep vein thrombosis; UTI, urinary tract infection; UEBMI, Urban Employee Basic Medical Insurance; URBMI, Urban Resident Basic Medical Insurance; NCMS, New Cooperative Medical System.

**Supplementary Table 4. Multivariate analysis for factors associated with hospitalization costs for immobile patients with** **stroke.**

|  | **Total^1)^（n=4716）** | | **Hemorrhagic^2)^（n=1573）** | | **Ischemic^3)^（n=3143）** | |
| --- | --- | --- | --- | --- | --- | --- |
|  | **B** | **SE** | **B** | **SE** | **B** | **SE** |
| constant | -15425.23* | 1185.55 | -32229.38* | 3939.29 | -11583.29* | 1029.58 |
| LoS | 1435.76* | 48.53 | 1183.95* | 147.76 | 1391.41* | 48.35 |
| Previous surgery | 25707.62* | 1573.24 | 30524.34* | 2798.87 | 19936.57* | 1833.14 |
| Hospital level | 25918.73* | 1270.08 | 45490.24* | 3630.41 | 20851.95* | 1145.30 |
| Previous ICU admission | 18932.86* | 1631.18 | 18562.96* | 3051.55 | 20012.31* | 1815.94 |
| Urethral invasive operation | 11917.18* | 1342.77 | 12211.79* | 2568.11 | 10338.59* | 1454.75 |
| Invasive ventilation therapy | 20361.28* | 2193.23 | 15288.83* | 3670.33 | 27413.61* | 2665.64 |
| Respiratory invasive operation | 36370.48* | 4474.34 | 38566.54* | 7442.69 | 30398.73* | 5639.84 |
| Pneumonia | 11114.14* | 1990.73 | - | - | 10960.55* | 2085.26 |
| DVT | - | - | - | - | 30242.50* | 5954.05 |
| Duration of immobility | - | - | 930.61* | 186.11 | - | - |
| Stroke subtype (hemorrhagic stroke) | 5660.20* | 1286.94 | - | - | - | - |

1) Adjusted R^2^=0.512; 2) adjusted R^2^=0.536; 3) adjusted R^2^=0.421.

* *P*<0.001

Abbreviations: LoS, length of stay; ICU, intensive care unit; SE, standard error; DVT, deep vein thrombosis.
